# Qualitative research and pain: Current controversies and future directions

**DOI:** 10.1080/24740527.2020.1809201

**Published:** 2020-09-24

**Authors:** Perri R. Tutelman, Fiona Webster

**Affiliations:** a Department of Psychology and Neuroscience, Dalhousie University, Halifax, Nova Scotia, Canada; b Centre for Pediatric Pain Research, IWK Health Centre, Halifax, Nova Scotia, Canada; c Arthur Labatt Family School of Nursing, Western University, London, Ontario, Canada

**Keywords:** pain, qualitative research, evaluation, quality, sample size, arts-based, phenomenology, grounded theory

## Abstract

Much of what we know about the meaning and experience of pain has been facilitated through qualitative research. However, qualitative inquiry continues to be underrepresented in the pain literature relative to quantitative approaches. In this Commentary and Introduction to the Special Issue on Qualitative Research and Pain, we present a collection of high-quality, cutting-edge qualitative studies in pain that highlight theoretical and methodological advancements in the field. The articles included in this Special Issue feature a range of designs (e.g., grounded theory, phenomenology, qualitative description), methods of data collection (e.g., interviews, object elicitation, photovoice), and populations (e.g., immigrant women, individuals with heart disease). Throughout this Commentary we also address three common controversies regarding the quality of qualitative research and the stance we took on them for the Issue. These primarily deal with the procedure-related issues of sample size, generalizability, and saturation. We discuss how a more substantive-centered approach to evaluation—that is, an approach that considers the methodological and theoretical significance of the work—is crucial for advancing qualitative research in pain.

## RÉSUMÉ

Une grande partie de ce que nous savons sur la signification et l’expérience de la douleur a été facilitée par la recherche qualitative. Cependant, l’enquête qualitative continue d’être sous-représentée dans la littérature sur la douleur en ce qui concerne les approches quantitatives. Dans ce commentaire et introduction au numéro spécial sur la recherche qualitative et la douleur, nous présentons un recueil de haute qualité d’études qualitatives de pointe sur la douleur qui mettent en évidence les avancées théoriques et méthodologiques dans le domaine. Les articles inclus dans ce numéro spécial présentent une gamme de conceptions (p. ex., la théorie ancrée, la phénoménologie, la description qualitative), de méthodes de collecte de données (p. ex., les entretiens, l’élicitation d’objets, le photovoice) et de populations (par exemple, les femmes immigrantes, les personnes atteintes de maladie cardiaque). Tout au long de ce commentaire, nous abordons également trois controverses courantes concernant la qualité de la recherche qualitative et la position que nous avons adoptée à leur égard pour ce numéro. Celles-ci traitent principalement de problèmes liés à la procédure, notamment la taille de l’échantillon, la généralisabilité et la saturation. Nous discutons de la façon dont une approche de l’évaluation davantage centrée sur le fond - c’est-à-dire une approche qui tient compte de la signification théorique et méthodologique des travaux - est primordiale pour faire avancer la recherche qualitative sur la douleur.

## Introduction

We are delighted to present the *Canadian Journal of Pain* Special Issue on Qualitative Research and Pain. As qualitative researchers in the field, we undertook the Special Issue to highlight the contributions that qualitative methods can offer to pain research. Much of what we know about the meaning and experience of pain, including understanding the experience of providers,^1^ has been facilitated through qualitative research conducted over several decades. Through the robust examination of personal narratives, institutional processes, and lived experiences, qualitative research has advanced our understanding in areas such as the multidimensional experience of living with pain,^2–5^ barriers to evidence-based pain care,^6–8^ and approaches to pain education^9,10^ in populations across the life span. Despite the strengths of qualitative research and its contributions to the study of pain, qualitative inquiry continues to be underrepresented in the pain literature relative to quantitative approaches.

In this Commentary, we take the opportunity to address common biases and misconceptions of qualitative research and to highlight the approach we took for the Special Issue. A complete list of misconceptions, and a full explanation of why we believe they are problematic, is beyond the scope of this Commentary, and these have been discussed elsewhere.^11–14^ However, we wish to briefly address our position on key issues to facilitate the development and publication of high-quality qualitative research in pain. Of note, we aim to discuss qualitative research within its own paradigm, epistemologies, and ontologies.

## Current Controversies

*Qualitative research* is an umbrella term to describe approaches to data collection and analysis that rely on largely language- and/or arts-based data. Language-based data is often conceptualized as transcripts of semistructured interviews between an interviewer and interviewee; however, it can take other forms, such as the compilation of historical documents, written story completion, or comments posted by users on social media. An innovative use of language-based data in qualitative pain research is demonstrated, in this Special Issue, by Toye et al.,^15^ who analyzed comments posted in response to a YouTube video on chronic pain. Two of the multiple approaches to arts-based qualitative research are also illustrated in the present Special Issue. For instance, Woodgate et al.^16^ explored the meaning of pain for youth with anxiety by analyzing photos they took of objects and people, and O’Keefe-McCarthy et al.^17^ investigated individuals’ journeys recognizing pain as a symptom of cardiovascular disease by interpreting participants’ thematic poetry and visual art. Language- and arts-based data can be used in unison to provide a comprehensive description of various phenomena.

Qualitative research is not a homogeneous category. There are various methodological approaches to qualitative research, including long-standing traditions such as grounded theory,^18^ ethnography,^19^ phenomenology,^20,21^ and qualitative description,^22^ as well as newer designs such as arts-based approaches.^23^ Each of these approaches have evolved over time, have many variations, and emerge from their own historical context, epistemological position, and methodological techniques. The guiding qualitative methodology (distinct from the methods such as focus groups or interviews) should be identified in every study because this sets the groundwork for congruence across all other aspects of the design, including the analytic approach. Partially due to this complexity, there is much debate in the field of qualitative research surrounding what constitutes “good” qualitative research (e.g., rigor) and how to evaluate the “goodness” of qualitative studies.^11,13,24,25^

Historically, approaches to the evaluation of research quality, derived from positivist research paradigms, often focused on procedural-centered criteria that relate to methods rather than to what Eakin and Mykhalovksi^11^ refer to as substantive-centered criteria relating to methodology and theory. In line with emerging best practices,^24^ we believe that procedural criteria (e.g., appropriateness of methods) should be used to guide one’s understanding of the substantive criteria (e.g., analytic content), not as a sole indicator of the quality of the work. For this reason, we describe three common misconceptions regarding the quality of qualitative research, which primarily deal with procedure-related issues: sample size, generalizability, and saturation. We discuss how a more substantive-centered approach to evaluation is crucial for advancing qualitative research in pain.

One of the most widely cited criticisms of qualitative research is the relatively small sample sizes associated with these approaches.^12,26^ We argue that small sample sizes are not a weakness of qualitative research but rather a core characteristic of many qualitative methodologies, given that the data are intended to provide rich and deep exploration rather than broad surveys of phenomena. As illustrated in the Special Issue, Dagg et al.^27^ show how small samples can be a strength by facilitating an in-depth idiographic approach to understanding the experience of pain that may not be accessible by the adoption of a more nomothetic approach. Dagg et al.^27^ explored the experiences of ten parents managing their children’s postoperative pain after discharge home from the hospital. Using an interpretive phenomenological analytic design, the authors provided rich, in-depth descriptions of the logistical, emotional, and relational challenges faced by parents providing postoperative pain care at home that provides important insights for both clinicians and researchers designing interventions. Sole judgment on the quality of a qualitative study based on the procedure-related criterion of sample size disregards the substantive contribution that a study may provide.

Sample size in turn is related to the issue of generalizability, long held to be a marker of impact and usability in research studies. This is perhaps the strongest criticism that has been leveled against qualitative research: that its findings are not representative of the population from which the study sample was derived. Qualitative researchers rely on the concept of transferability rather than generalizability to describe how their findings can generate theoretical or conceptual insights that can be applied more broadly and is considered by many a key criterion of rigor.^14,28^

We illustrate the point of transferability of qualitative findings using two articles in the Special Issue.^28,29^ Using a qualitative descriptive methodology, Dale et al.^28^ interviewed recently discharged patients about their procedural oral pain experiences while mechanically ventilated in the intensive care unit (ICU). Using an object elicitation approach, the authors found that patients recalled significant pain and distress related to oral care in the ICU but that, because of ventilation, were unable to self-report this pain. Participants described that their attempts to communicate their pain (e.g., grimacing, biting down) were often misinterpreted as agitation or uncooperative behavior. These data provide conceptual insight regarding the oral pain experienced by patients in the ICU and highlight troublesome gaps in ICU clinicians’ pain knowledge. The findings from this study are transferrable to the growing literature on the development of pain education frameworks in various disciplines such as medicine, nursing, and dentistry.^30–32^

Similarly, Mustafa et al.^29^ explored the lived experience of chronic pain in immigrant Indian-Canadian women using a phenomenological design. The authors found that immigrant Indian-Canadian women’s experiences of pain are influenced by several unique sociocultural factors (e.g., gender roles, work burden, lack of social support), which in turn impact pain expression, openness to treatment, and the consequences of pain on the family. The implications of this study are transferable to numerous other populations and settings, demonstrating the importance of considering the cultural values and norms of patients with chronic pain.

Finally, the term *saturation* and its meaning remain contentious in the qualitative literature.^25,33^ Indeed, saturation is a central component of certain qualitative approaches (e.g., grounded theory^18^); however, it is unhelpful, unachievable, and in direct opposition to the particular epistemology associated with certain designs and methods, including phenomenology^26^ and reflexive thematic analysis.^34^ By example, Tabeefar et al.^35^ used thematic saturation as a construct to guide sampling adequacy in their study of community pharmacists’ experiences with chronic pain, which was guided by the principles of grounded theory. Conversely, and in line with phenomenological inquiry, the studies by Dagg et al.,^27^ Mustafa et al.,^29^ and Woodgate et al.^16^ focused on obtaining rich personal accounts and describing the similarities and differences in experiences across participants. A one-size-fits-all approach to evaluating qualitative studies, as is often done by non-qualitative experts, disregards the core epistemological and ontological underpinnings of the various qualitative designs.

We argue that the misapplication of procedure-related evaluation criteria (such as sample size, generalizability, and saturation) to qualitative studies by nonqualitative experts, and even some who may be familiar with qualitative approaches, has led to inaccurate appraisals about the rigor and significance of qualitative research, and thus, exclusion of high-quality qualitative studies from top pain journals. In order to generate and retain high-quality qualitative research in pain, we must offer enhanced training opportunities for those interested in engaging with these methods. We must also increase the number of journal reviewers and editors who are competent and knowledgeable in substantive issues related to qualitative methods to ensure that high-quality qualitative research is not turned away from medical journals and that misunderstandings about qualitative research do not continue to be perpetuated. To this end, we conceived of the present Special Issue as a way of highlighting novel and innovative qualitative research that is being conducted in the field of pain.

## The Special Issue on Qualitative Research and Pain

We received a tremendous response to the call for manuscripts for the *Canadian Journal of Pain* Special Issue on Qualitative Research and Pain. This high level of interest demonstrates the strong appetite for qualitative research in pain as well as the large number of qualitative studies being conducted. Due to the volume of submissions, we were only able to accept the highest quality manuscripts, which we defined as involving both a strong substantive approach (e.g., novel contribution, in-depth theoretical or conceptual insight) and that demonstrated high rigor according to appropriate standards such as congruence.

This carefully curated collection includes high-quality, cutting-edge empirical qualitative studies in pain that highlight theoretical and methodological advancements in the field. The studies included in this Special Issue employ a range of qualitative methodologies, including grounded theory,^35^ phenomenology,^16,27,29^ qualitative description,^28^ and arts-based approaches.^15,17^ The collection of studies also demonstrates the array of methods of data collection possible in qualitative research, including both traditional (e.g., one-on-one interviews^27,29,35^) and novel (e.g., photovoice,^16^ object elicitation,^28^ social media posts^15^) methods. Finally, the Special Issue articles illustrate the capacity of qualitative research to capture the meaning and experience of pain in diverse populations, including children with anxiety,^16^ critically ill patients,^28^ pharmacists working with individuals with chronic pain,^35^ immigrant women,^29^ parents of children who have undergone surgery,^27^ individuals with heart disease,^17^ and those with various forms of chronic pain.^15^

## Conclusion

We hope that this Special Issue offers an introduction to the diversity and importance of qualitative findings to our collective understandings of pain. The articles in the Special Issue are a sampling of the many qualitative methodologies that can be applied to pain research. Some methodologies (e.g., ethnography) are not represented but are also valuable for the investigation of pain. We strongly encourage researchers interested in understanding the experiences, meaning, or organization of pain to seek out the vast array of high-quality qualitative research available.
